# Boundary effects influence velocity of transverse propagation of simulated cardiac action potentials

**DOI:** 10.1186/1742-4682-2-36

**Published:** 2005-09-06

**Authors:** Nicholas Sperelakis, Bijoy Kalloor, Lakshminarayanan Ramasamy

**Affiliations:** 1 Dept. of Molecular & Cellular Physiology, University of Cincinnati College of Medicine, Cincinnati, OH 45267-0576, USA; 2 Dept. of Electrical Computer Engineering and Computer Science, University of Cincinnati, College of Engineering, Cincinnati, OH 45221, USA

**Keywords:** Propagation of cardiac action potential, transverse propagation velocity, PSpice simulations, edge/boundary effects, electric field transmission of excitation.

## Abstract

**Background:**

We previously demonstrated that transverse propagation of excitation (cardiac action potentials simulated with PSpice) could occur in the absence of low-resistance connections (gap – junction channels) between parallel chains of myocardial cells. The transverse transmission of excitation between the chains was strongly dependent on the longitudinal resistance of the interstitial fluid space between the chains: the higher this resistance, the closer the packing of the parallel chains within the bundle. The earlier experiments were carried out with 2-dimensional sheets of cells: 2 × 3, 3 × 4, and 5 × 5 models (where the first number is the number of parallel chains and the second is the number of cells in each chain). The purpose of the present study was to enlarge the model size to 7 × 7, thus enabling the transverse velocities to be compared in models of different sizes (where all circuit parameters are identical in all models). This procedure should enable the significance of the role of edge (boundary) effects in transverse propagation to be determined.

**Results:**

It was found that transverse velocity increased with increase in model size. This held true whether stimulation was applied to the entire first chain of cells or only to the first cell of the first chain. It also held true for retrograde propagation (stimulation of the last chain). The transverse resistance at the two ends of the bundle had almost no effect on transverse velocity until it was increased to very high values (e.g., 100 or 1,000 megohms).

**Conclusion:**

Because the larger the model size, the smaller the relative edge area, we conclude that the edge effects slow the transverse velocity.

## Introduction

Computer simulation of the propagation of impulses in cardiac muscle shows that the electric field generated in the narrow junctional clefts when an action potential occurs at the prejunctional membrane depolarizes the postjunctional membrane to threshold [1]. Thus, the postjunctional cell is excited after a brief delay at the junction and propagation in cardiac muscle is saltatory. We have modeled APs in this tissue using the PSpice program for circuit design and analysis, and we have corroborated earlier reports that the EF developed in the junctional cleft is sufficiently large to allow transfer of excitation to the contiguous cell without the requirement for a gap-junction [2-6]. To date, however, we have only used small-sized models for these simulation studies.

When our paper on transverse propagation of cardiac action potential (APs) simulated by PSpice in a 5 × 5 model [4,5] was reviewed by the journal, one unanswered question was whether edge (boundary) effects were important. The purpose of the present study was to address this question. To do this, we expanded the model to a 7 × 7 size (7 parallel chains of 7 cells each). Thus, we could compare transverse velocity in 2-dimensional models of 4 sizes: 7 × 7, 5 × 5, 3 × 4, and 2 × 3. It was essential that all circuit parameters were the same in all four models. It was found that the larger the model, the faster the transverse velocity of propagation, up to a presumed saturation point.

## Methods

**Figure 1:**
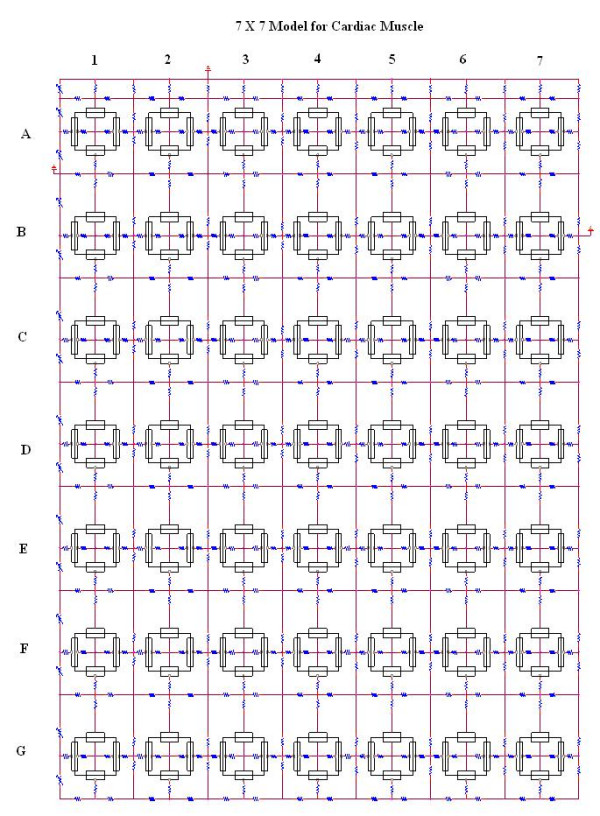
**7 × 7 Model for Cardiac Muscle: **Block diagram of the 7 × 7 model for cardiac muscle. These were 7 parallel chains (A-G) of 7 cells each (1–7). The cells longitudinally were separated by high-resistance cell junctions, with a radial junctional cleft resistance (R_jc_) of 25 MΩ (50 MΩ/2). The parallel chains were separate and also not connected by gap-junction channels. The longitudinal resistance of the interstitial space between the parallel chains (R_ol2_) had values of 200 KΩ and 500 KΩ. Both ends (termination) of the tissue bundles were connected by transverse resistances (R_BT_); the value was 1.0 KΩ, but much higher values were tested. There was only little effect of varying R_BT _over a wide range, until very high values of 500 MΩ were inserted. As shown, there were 4 basic units for each cell: two for the surface membrane (one facing upwards and one downwards) and one for each of the two junctional membranes. The resistive and capacitive elements of the surface membrane and junctional membranes were prorated based on the relative areas. Electrical stimulation (rectangular current pulse of 0.5 nA and 0.5 ms) was applied intracellularly, either to the entire chain (A or G) or to the first cell only of these chains (cell A1 or cell G1).



The detailed methods and circuit parameters used for cardiac muscle were described previously [2,4,5]. As shown in Figure 1 (7 × 7 model), there were two surface membrane units in each cell (one facing upwards and one inverted) and one unit for each junctional membrane (intercalated disk). The values for the circuit parameters used (standard conditions) are listed in Table 1 (footnote) for both the surface units and the junctional units. Under standard conditions, R_ol2 _was 500 KΩ, R_or2 _was 100 Ω, and R_jc _was 25 MΩ (50 MΩ ÷ 2). The R_ol2_/ R_or2 _ratio of 5000 was calculated from the equation relating absolute resistance to the resistivity of the interstitial fluid (ρ) (50 Ω – cm) and the distance (L) and cross-sectional area (A_x_);

**Table 1:** Transverse Propagation Velocity (antegrade (A) and retrograde(R)) of Simulated Cardiac Action Potentials in 2-D Sheets at a R_ol2 _of 500 KΩ.

Model Size		Stimulations	No. of Chains Responding	TPT ms	Transv. Velocity cm/sec
7 × 7	**A**	Entire A Chain	7	1.2	8.0
		Cell A1 Only	7	1.5	6.4
	**R**	Entire G Chain	7	1.2	8.0
		Cell G1 Only	6 (A failed)	1.5	5.4
5 × 5	**A**	Entire A Chain	5	1.6	4.0
		Cell A1 Only	5	1.7	3.8
	**R**	Entire E Chain	5	1.7	3.8
		Cell E1 Only	5	1.8	3.6
3 × 4	**A**	Entire A Chain	3	1.0	3.2
		Cell A1 Only	3	1.1	2.9
	**R**	Entire C Chain	3	1.2	2.7
		Cell C1 Only	3	1.2	2.7
2 × 3	**A**	Entire A Chain	2	0.7	2.3
		Cell A1 Only	2	0.8	2.0
	**R**	Entire B Chain	2	0.9	1.8
		Cell B1 Only	2	0.9	1.8

A = antegrade R = retrograde

Circuit Parameters: R_BT _= 10 KΩ R_jc _= 25 MΩ (50 MΩ/2); C_j _= 16 pF; C_s _= 0.2 pF

To improve the performance of the 7 × 7 model (for R_ol2 _of 500 KΩ), R_jc _was decreased slightly to 24.5 MΩ (49.0 MΩ/2).

The myocardial cell was assumed to be a cylinder 150 μm long and 16 μm in diameter. The cell capacitance was assumed to be 100 pF, and the input resistance to be 20 MΩ. A junctional tortuosity (interdigitation) factor of 4 was assumed for the cell junction [1,2]. The junctional cleft potential (V_jc_) is produced across R_jc_, the radial resistance of the narrow and tortuous junctional cleft. The junctional cleft contained two longitudinal resistances of 7Ω each and two radial resistances (R_jc_) of 50 MΩ each in parallel.

The tortuosity factor does not interact with the packing factor. The tortuosity factor concerns the complex interdigitation of contiguous cells longitudinally (end-to-end), whereas the packing factor deals with how closely the cell chains are packed transversely (or radially) within a tissue bundle. The value assigned to R_ol2 _reflects the closeness of this packing. The value assigned to Rjc reflects the thickness of the junctional gap (end-to-end) and the tortuosity factor.

The circuit used for each unit was kept as simple as possible, using only those ion channels that set the resting potential (RP) and predominate during the rising phase of the AP. We wanted only to inscribe the rising phase of the APs to study propagation in the 2-dimensional sheet. The RP was -80 mV and the overshoot potential was +30 mV (AP amplitude of 110 mV). Transverse propagation velocity was calculated from the measured total propagation time (TPT) (measured as the difference between when the APs of the first cell and last cell crossed -20 mV) and cell width (number of chains minus one gives the number of transverse junctions traversed).

Because the PSpice program does not have a V-dependent resistance to represent the increase in conductance for Na^+ ^ions in myocardial cells during depolarization and excitation, this function was simulated by a V-controlled current source (our "black-box") in each of the basic circuit units. The current output of the black-box at various membrane voltages was calculated assuming a sigmoidal relationship between membrane voltage and resistance over the range of -60 mV to -30 mV. The V values used in the GTABLE were those recorded *directly across *the membrane. The excitability of the basic units was the same as in our previous papers, i.e., it was set at the moderate level [6].

The upper chain of cells was assumed to be bathed in a large volume of Ringer solution connected to ground. The external resistance (R_o_) of this fluid was divided into two components: a radial resistance (R_or_) and a longitudinal resistance (R_ol_). The longitudinal resistance value between the chains (R_ol2_) was increased over a wide range to reflect closer packing of parallel chains into a bundle of fibers (Fig. 1). The transverse resistance of the interstitial fluid space (R_or2_) was found to have almost no effect on the transverse velocity. The cells in each chain were not interconnected by low-resistance pathways (gap-junction channels), so that transmission of excitation from one cell to the next had to be by the electric field (EF) developed in the narrow junctional cleft. In our previous papers, we presented a number of references demonstrating that propagation velocity is slowed only slightly in the absence or paucity of gap junctions [e.g., see refs [1,3] and [7]]. There were seven parallel chains (chains A-G) of seven cells each in the 7 × 7 model. The block diagrams and detailed circuitry for the other models (5 × 5; 3 × 4; 2 × 3) were previously published [4,5]. The ends of each chain had a bundle termination resistance (R_BT_) of 1.0 KΩ to mimic the physiological condition. However, variation of R_BT _over a wide range had almost no effect, until very high values of about 500 MΩ were inserted.

Electrical stimulation (rectangular current pulses of 0.50 nA and 0.50 ms duration) was applied to the inside of either the first cell of chain A (cell A1) or simultaneously to all cells of the A-chain. For retrograde propagation, stimulation was applied either to cell G1 or to all cells of the G-chain. For some measurements, the V-recording markers were placed on only one chain at a time. To minimize confusion, the voltage was recorded from only one surface unit (upward-facing) in each cell.

## Results

The results to be illustrated here will be from the 7 × 7 model only, because this model is new. However, the results from the smaller models (5 × 5, 3 × 4, 2 × 3), previously published, are summarized in Table 1. Thus, Table 1 enables transverse propagation velocities to be compared in four models differing in size but with identical circuit parameters used in the basic units. Through this comparison, it can be ascertained whether edge (boundary) effects are important in transverse propagation.

**Figure 2:**
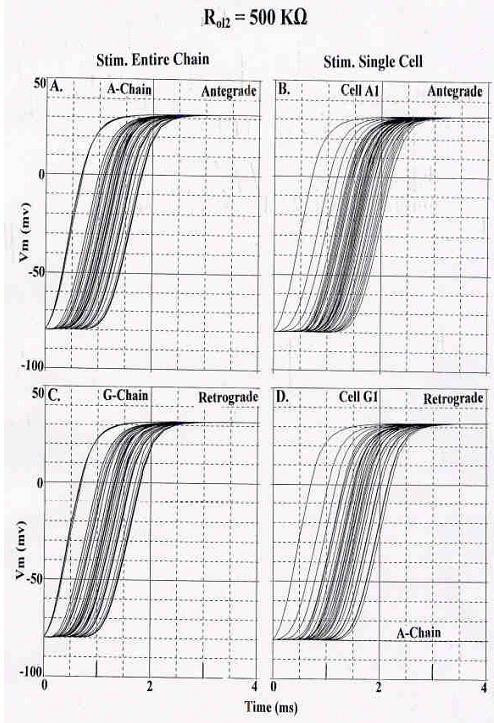
Rising phases of the simulated APs recorded from the 7 × 7 model for cardiac muscle when R_ol2 _was 500 KΩ. **A-B: **Antegrade propagation. **A: **Stimulation of the entire A chain. No chains failed, and TPT was short. Many traces overlap. **B: **Stimulation of only cell A1. Again, there were no failures. TPT was prolonged (compare to Panel A). **C-D: **Retrograde propagation. **C: **Stimulation of entire G-chain. No failures occurred. TPT was about the same as in panel A (for orthodromic). **D: **Stimulation of only cell G1. The last chain (A) failed.

In the 7 × 7 model, with all circuit parameters having the standard values, including R_ol2 _of 500 KΩ, either the entire A-chain was stimulated simultaneously (Fig. 2A) or only cell A1 was stimulated (Fig. 2B) (for antegrade propagation). Since the circuit was symmetric, the terms "antegrade" and "retrograde" are arbitrary, and are used simply to denote direction of propagation. For retrograde propagation, the entire G-chain was simultaneously stimulated (Fig. 2C) or only cell G1 was stimulated (Fig.2D). As can be seen, the A-chain failed in the retrograde (antidromic) direction (Fig. 2D) when a single cell was stimulated. However, there were no failures when the entire G-chain was stimulated (Fig. 2C). Increasing R_ol2 _caused fewer chains to fail, and propagation velocity was increased substantially (TPT decreased). Thus, retrograde propagation is not always identical to the antegrade propagation, though it is always very close. Since the PSpice program generates a netlist error indicating the presence of any floating node, we suggest that the aberrant retrograde propagation behavior is a limitation in the PSpice computational algorithm rather than a property of the model. Activation maps would have revealed the patterns in more detail, but the software for obtaining such maps was not available when these experiments were performed.

Table 1 summarizes all these data, not only for the 7 × 7 model, but also for the smaller models of 5 × 5, 3 × 4, and 2 × 3. These data include antegrade (A) and retrograde (R) propagation, with stimulation of the entire chain or single cell only, for the R_ol2 _value of 500 KΩ. As can be seen, the calculated transverse propagation velocities were highest in the large 7 × 7 model and slower in the smaller models. This was true for all values of R_ol2_. Transverse propagation velocities were faster at R_ol2 _of 500 KΩ than at 200 KΩ.

**Figure 3:**
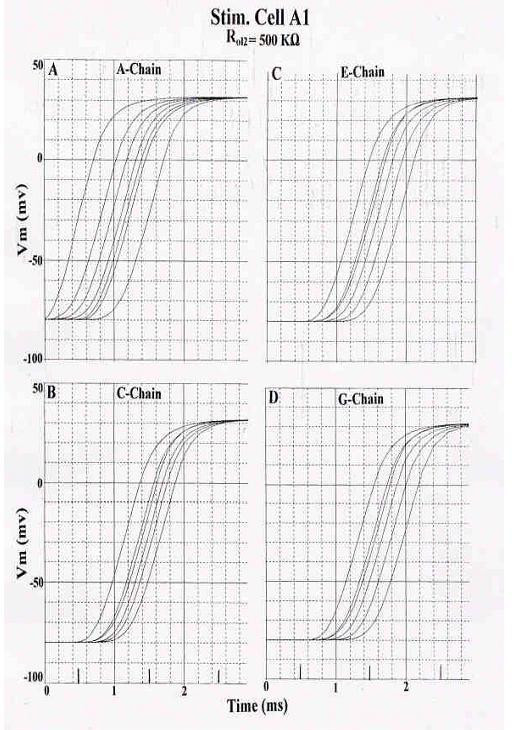
Recordings of the APs from one chain at a time, so that the transverse spread of excitation can be more clearly seen. 7 × 7 model of cardiac muscle. Standard conditions for all circuit parameters; R_ol2 _was 500 KΩ. Stimulation was applied to cell A1 (first cell of A-chain). All 49 cells responded. To reduce the number of panels, records from every other chain are illustrated. **A: **Records from the A- chain. **B: **Records from the C- chain. **C: **Records from the E- chain. **D: **Records from the G- chain. As can be seen, the stimulated A-chain responded earlier, followed by C-, E-, and G-chains. But there was some overlap between the traces from the various chains, indicating that transverse propagation between chains occurs simultaneously with longitudinal propagation within each chain.

To help clarify how propagation spreads through the 7 × 7 model, recordings were made from one chain at a time when stimulation was applied to cell A1 (Fig. 3). Records from selected chains are illustrated to allow appreciation of the time sequence of firing of the various chains. The stimulated chain always begins to respond first, but there is some overlap of firing from the adjacent chain. Thus, transverse propagation between chains occurs simultaneously with longitudinal propagation within each chain when only one cell is stimulated. When the middle chain (D-chain) of the network was stimulated, transverse spread of excitation occurred simultaneously in both directions (not illustrated). Transverse spread occurs at multiple points along the length of the chain.

## Discussion

**Figure 4:**
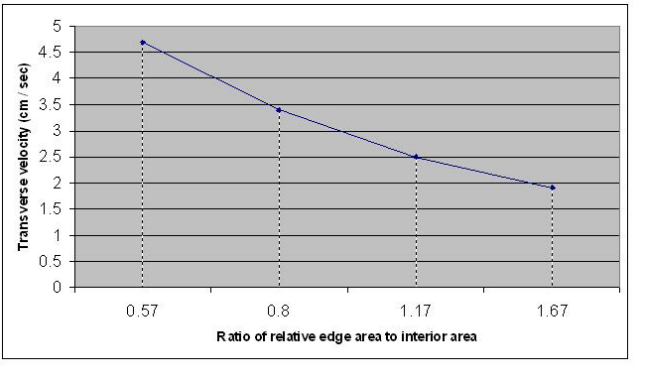
Plot of transverse velocity of propagation of the simulated cardiac action potentials as a function of the ratio of relative edge area to interior area (see Table 2).

The present results, comparing the velocities of transverse propagation (θ_tr_) in cardiac models differing in size but with identical circuit parameters, demonstrate that edge/ boundary effects have a strong action on transverse velocity (Fig. 4). θ_tr _was slowest in the smaller models and fastest in the larger models. In our new large 7 × 7 model, θ_tr _was about double the value in the 5 × 5 model (at R_ol2 _of 500 KΩ) (Table 1). Since the larger the model, the less the relative area of edges and the faster the propagation velocity, this means that edges must slow down θ_tr_.

In fact, θ_tr _is almost inversely proportional to the ratio of edge to interior areas for the four models compared in the present study (Table 2). This table relates the ratio of velocities to the inverse ratio of the relative edge area (or volume), using the 7 × 7 model as the base for comparison (A/Y for velocity and Y/A for relative edge area). The comparisons are: 1.38 vs 1.40; 1.88 vs 2.05; and 2.47 vs 2.93 (for R_ol2_200 KΩ). These comparisons are strikingly close. Comparisons for a R_ol2 _of 500 KΩ were also close: 2.00 vs 1.40, 2.50 vs 2.05, and 3.48 vs 2.93 (Table 1). The data for R_ol2 _of 200 KΩ are plotted in Figure 4.

**Table 2:** Comparison of the inverse ratios of the relative edge areas of the various-sized cardiac models with the ratio of the transverse propagation velocities (θ_tr_)

	Model Size	Ratio of relative edge area to interior area	θ_tr _cm/sec	Velocity A/Y	Area Y/A
A	7 × 7	28/49 = 0.57	4.7	--	--
B	5 × 5	20/25 = 0.80	3.4	1.38	1.40
C	3 × 4	14/12 = 1.17	2.5	1.88	2.05
D	2 × 3	10/6 = 1.67	1.9	2.47	2.93

Orthodromic direction; stim of entire A-chain; values for R_ol2 _of 200 KΩ.

Y equals value of B, C, or D.

The equation relating these parameters is:

Where a single asterisk superscript (*) denotes the values for the smaller model compared to the referral 7 × 7 model (denoted by double asterisk**).

This means that one can predict the transverse propagation velocities in yet-larger models. For example, in a 10 × 10 model, θ_tr _should be approximately 6.70 cm/s (if compared with the 7 × 7 model) or 6.80 cm/s (if compared with the 5 × 5 model). From the equation given in the footnote of Table 2:



Hence, the two calculations are in close agreement. However, this relationship between transverse velocity and the inverse of the relative edge area probably saturates and levels off at some point, i.e., a maximum θ_tr _is reached. In the intact heart, the velocity of transverse propagation is difficult to measure accurately because of the complicated geometry of bundles, but estimates that θ_tr _is about 1/5^th ^to 1/10^th ^that of θ_lo _(longitudinal velocity) have been given (see references given in ref 1). If θ_lo _is taken to be 0.40 m/s, then θ_tr_should be between 4.0 and 8.0 cm/s. Thus, the values calculated in the present simulations are in good agreement with physiological measurements.

The ratio of propagation velocities, longitudinal (θ_lo_) to transverse (θ_tr_), is almost what is expected based on the cell geometry (cylinder 150 μm long and 16 μm wide). These dimensions would predict a θ_lo _/ θ_tr _ratio of 9.4 (150/16), provided that the longitudinal and transverse transfer function are equal (i.e, the delay time at the two types of junctions were equal). If the average cell length were taken to be only 100 μm, then the θ_lo _/ θ_tr _ratio would be 6.3. Thus, for a θ_lo _value of 40 cm/s and a θ_lo _/ θ_tr _ratio of 7.9 (average of 9.4 and 6.3), then θ_tr _would be 5.1 cm/s, which is close to the value of 4.7 cm/s measured in the 7 × 7 model (for R_ol2 _of 200 KΩ). However, it has been reported that the anisotropic conduction velocity observed in the heart is not a result of cell geometry [8].

Another observation in the large 7 × 7 model is that some chains distal to the point of stimulation failed when R_ol2 _was only 200 KΩ. Such failures did not occur when the model was smaller (e.g., 5 × 5). Failure of distal chains occurred in both the orthodromic and antidromic directions, but was greater in the antidromic direction. However, increasing R_ol2 _to 500 KΩ allowed all chains to respond, with the exception of failure of one chain (the most distal A-chain) in the retrograde direction (Table 1). Therefore, in the largest model, there is an increase in probability of failure of one or more distal chains.

Although we don't know the mechanism for this effect, we may speculate about two possibilities. First, if some current leaked out at the ends of each chain, then less current would be available for downstream depolarization. Second, if the phenomenon of reflection occurred at the longitudinal edge of the last chain (G), then this would act to slow the transverse velocity. Thus, both of these mechanisms may be involved in explaining why transverse propagation was faster in the larger models.

In summary, the present results using our enlarged 7 × 7 model for cardiac muscle, with comparisons with our prior smaller models, demonstrate that edge effects are important in determining the transverse velocity of propagation, when all circuit parameters are identical. θ_tr _increased with the inverse of the ratio of the relative edge areas in the various-sized models. This relationship likely levels off at some point, such that a maximum velocity is reached. The transverse velocities measured in the largest model (7 × 7), and estimated for a 10 × 10 model, give values in the same range as the physiological values.
